# Mapping trends and hotspot regarding testicular torsion: A bibliometric analysis of global research (2000–2022)

**DOI:** 10.3389/fped.2023.1121677

**Published:** 2023-02-28

**Authors:** Shaowen Hu, Mingjie Guo, Yafei Xiao, Yang Li, Qingyang Luo, Zun Li, Chaoyang Zhu

**Affiliations:** ^1^ Department of Urinary Surgery, Huaihe Hospital of Henan University, Kaifeng, China; ^2^ Department of Thoracic and Cardiovascular Surgery, The First Affiliated Hospital of Henan University, Kaifeng, China; ^3^ Gastrointestinal Surgery, Huaihe Hospital of Henan University, Kaifeng, China

**Keywords:** acute scrotum, testicular torsion, ischemia reperfusion, orchiectomy, citespace

## Abstract

**Background:**

Testicular torsion is an acute scrotal disorder requiring immediate emergency treatment. Ischemic injury and reperfusion injury are important causes of oxidative stress and irreversible oxidative damage after testicular torsion. Although a large number of literatures have discussed the causes and treatment of testicular torsion, there is currently a lack of systematic exploration of the historical evolution of testicular torsion and the construction of a knowledge framework.

**Method:**

The Web of Science Core Collection was searched for studies on testicular torsion published between 2000 and 2022. The basic data of the literature were analyzed by using Excel and CiteSpace software.

**Result:**

A total of 1,007 publications on testicular torsion published were found in 64 countries between 2000 and 2022, with an increasing annual publication level. Early detection, early diagnosis and early treatment of testicular torsion had always been at the core of clinical practice, and the pathological cascade reaction of ischemic injury and ischemia-reperfusion injury after testicular torsion were also at the core of basic research. Emphasis had been placed on the development of protective drugs for ischemia and reperfusion after testicular torsion in various countries, regions and institutions.

**Conclusion:**

Over the past 20 years, the research on testicular torsion had been widely concerned. Hot topics in testicular torsion in recent years were ischemia-reperfusion injury, oxidative stress, rat, doppler ultrasonography, diagnosis and orchiectomy. This article may provide a useful resource for clinicians and basic researchers regarding testicular torsion.

## 1. Introduction

Testicular torsion, the twisting of the spermatic cord and its contents, is a urological emergency (1). The patient mainly had acute scrotal pain on the affected side, accompanied by nausea and vomiting (2). Strict physical examination can reveal the high-riding testis and absent cremasteric reflex (3). Color doppler ultrasonography can point out the reduction or even disappearance of the testicular blood flow signal (4). Once testicular torsion was considered, scrotal exploration was required to clarify the cause. The chance of testicular salvage after testicular torsion decreases with increasing degree of torsion and duration of symptoms (5). The optimal time for testicular salvage is within 6 h (6). Even immediate surgical treatment without preoperative imaging exploration was required to increase the success rate of testicular salvage (7). Although early surgical intervention increases the likelihood of testicular salvage, only 32% of testes can be salvaged, and almost all patients with testicular torsion undergoing salvage surgery experienced varying degrees of testicular atrophy postoperatively (8, 9). In addition, among boys undergoing testicular torsion surgery, the orchidectomy rate was 42%, and experimental data showed that children aged 1 to 9 years were at higher risk of orchidectomy (7, 10). Currently, testicular torsion had been shown to cause a long-term decline in sperm motility and a reduction in the total number of sperm, which caused great harm damaging to the families of children with testicular torsion (11). With the improvement of people's awareness of testicular torsion, the early detection, diagnosis and treatment of testicular torsion had been widely responded by people(12). Basic researchers were also actively establishing rat model to develop a variety of drugs to protect ischemia-reperfusion injury after testicular torsion, such as erythropoietin receptor agonists, antioxidants, calcium channel blockers, and inflammation modulators etc (13).

CiteSpace, a free tool often used for bibliometric analysis, may provide more insights than traditional literature reviews (14). We aimed to use CiteSpace to provide the historical evolution of testicular torsion and build its relevant knowledge map, then analyzed the main research countries, institutions, key articles and keywords, so as to understand its development in the past 20 years and predict its future direction, and finally provided ideas for further research.

## 2. Data collection and research methods

### 2.1. Data source and collection

On November 10, 2022, we searched the Web of Science Core Collection (WoSCC) for relevant articles in the field of testicular torsion from 2000 to 01–01 to 2022–11–01. SCI-EXPANDED, CPCI-S, CPCI-SSH, BKCI-S and BKCI-SSH were used as data sources, and the publication type was limited to “article”. The main search terms were set as “spermatic cord torsion”, “twisting of the spermatic cord”, “torsion of testicular cord”, “testicular torsion”, and “pediatric testicular torsion”. Detailed search strategies were described in the supplementary materials. The two authors (SH and MG) independently searched for relevant literature in the WoSCC database, then downloaded and saved the retrieved literature in the format of “full record with cited references” for literature screening, and finally obtained 1,007 literature as a sample for visual analysis.

### 2.2. Research methods

CiteSpace was a visualization software for bibliometric analysis based on the Java platform developed by Professor Chaomei Chen. It was an interactive analysis tool for analyzing basic indicators of literature including country, journal, institution, keyword and co-citation of references. It can capture keywords with strong citation references, and assist in the scientific visualization tasks (15). The CiteSpace software version used was 6.1.R3 (64-bit) in this study. The parameters set as follows: the date was set from January 2001 to November 2022 (each slice is 1 year), select all options in term source, select one node type at a time, and select TOP50 or TOP30 as the standard, and other setting items as default values. Nodes and linear connections were used to generate a visual knowledge map. Each node in the map represents an element to be analyzed, such as the cited countries, institutions, or authors. The size of the nodes represented the frequency of citations, and nodes of different colors represented different years. Linear connections between nodes were considered as co-occurrence or co-citation relationships, and the thickness of the line meant the strength of the relationship. The colors corresponded to the first co-occurrence or co-citation time of the nodes, and warmer colors represented the closer time.

Microsoft Office Excel 2016 (Microsoft, Redmond, Washington, United States) was used to process data and generate statistical charts.

## 3. Result

### 3.1. Quantitative analysis of basic information

#### 3.1.1. Annual growth trends in publications

**Figure 1:**
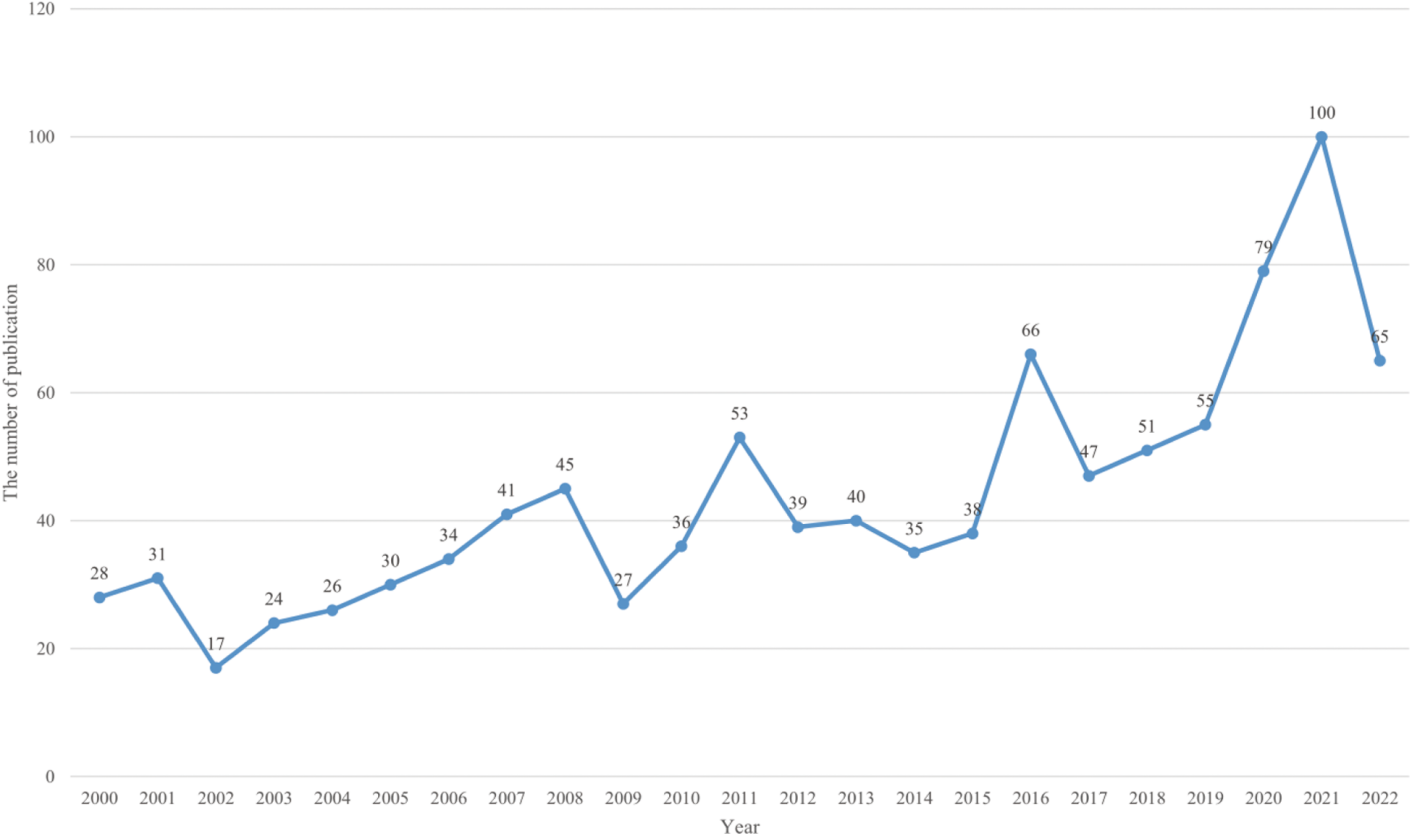
Time sequence of relevant papers on testicular torsion published from 2000 to 2022 in Web of science.

A search of the WoSCC database yielded a total of 1,007 articles on research in the field of testicular torsion published between January 2000 and November 2022. As shown in Figure 1, the number of published papers in 2002 was the least (*n* = 17, 1.69%), while the number of published publications in 2021 was the largest (*n* = 100, 9.93%) within the time limit of retrieval. In addition, the number of published documents in 2022 was 65, which slightly decreased compared with the previous year. This may be related to the fact that only 10 months of articles were counted in 2022, and the annual average number of published articles was 44. Overall, the average number of articles published annually showed an upward trend, except for a small decrease in some individual years between 2000 and 2021, indicating that this research was one of the current hot spots in urology and was still evolving.

#### 3.1.2. Country/region analysis

**Figure 2:**
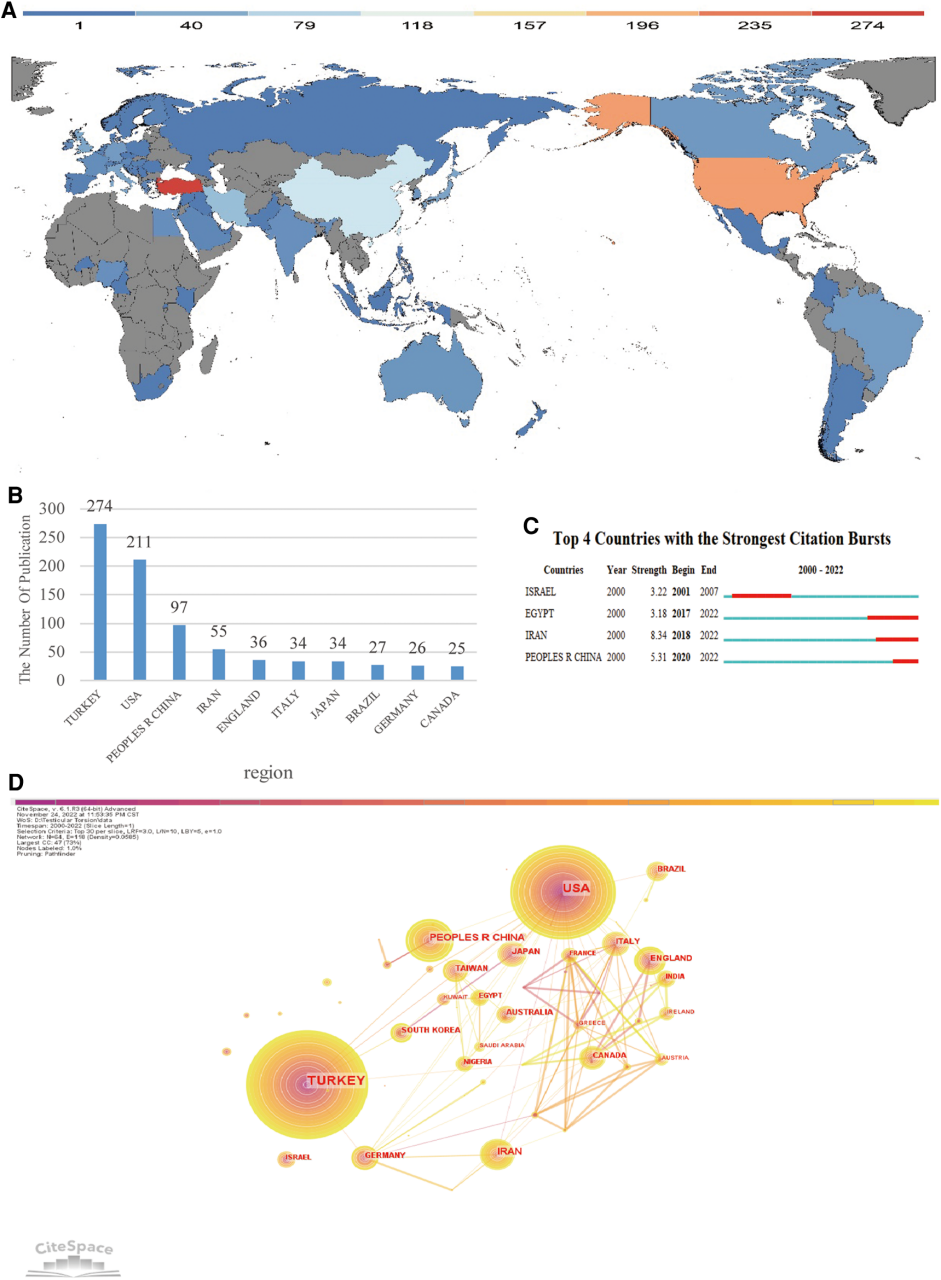
Analysis of countries engaged in research on testicular torsion. (**A**) The distribution of countries in terms of publications. (**B**) The top 10 most productive countries. (**C**) Countries with periods of burst from 2000 onward among the top 4 burst countries in articles related to Testicular Torsion. (**D**) Network diagram showing countries links.

According to the numbers of published literature, we drew the national distribution map and histogram shown in (Figure 2A) and (Figure 2B). The merged network consisted of 64 nodes and 118 linear connections. The nodes and linear connections between them revealed the national and regional cooperative relationships, respectively. In the past 20 years, 64 countries and regions had published a total of 1,007 documents. The top 10 countries (4 European countries, 3 Asian countries, 1 North American country and 1 South American country) published a total of 796 articles, accounting for 79.05% of the total articles. On the contrary, other countries and regions were less involved. The top 3 countries and regions were Turkey (*n* = 274, 27.21%), the United States (*n* = 211, 20.95%) and China (*n* = 74, 7.35%). Although Turkey had the largest number of published literatures, the United States had cooperated with more countries, such as Britain, France, Germany and Italy in Europe, Japan, China and Iran in Asia, as well as Australia. Comparatively speaking, China had cooperated with relatively few regions, mainly Belgium and Taiwan (China). According to the burst analysis shown in Figure 2C, the research on testicular torsion first became a hotspot in Israel during 2001–2007, and then since 2017, the research on testicular torsion had gradually increased in Egypt, Iran, and China as shown in Figure 2D.

#### 3.1.3. Institutional analysis

The ranking of institutional published volume is shown was Table 1, the top 3 were Tehran University Academy of Medical Sciences (*n* = 22, 2.18%), Karadeniz Technology University (*n* = 17, 1.69%) and Dicle University (*n* = 16, 1.59%). Tehran University Academy of Medical Sciences published the most papers, but the average year of publication was earlier; In recent years, the University of Health Sciences from Turkey (*n* = 14, 1.39%) had published more papers. Furthermore, Karadeniz Technology University and Dicle University, which ranked 2nd and 3rd, were both from Turkey, which illustrated that the research enthusiasm in Turkey on testicular torsion had been continuing.

**Table 1:** Ranking of the number of articles published by institutions.

Rank	Count	Year	Institutions
1	22	2005	Univ Tehran Med Sci
2	17	2001	Karadeniz Tech Univ
3	16	2003	Dicle Univ
4	14	2018	Univ Hlth Sci
5	13	2002	Ankara Univ
6	13	2007	Ataturk Univ
7	13	2002	Gazi Univ
8	13	2008	Gaziosmanpasa Univ
9	13	2004	Univ Messina
10	13	2000	Univ Virginia

**Figure 3:**
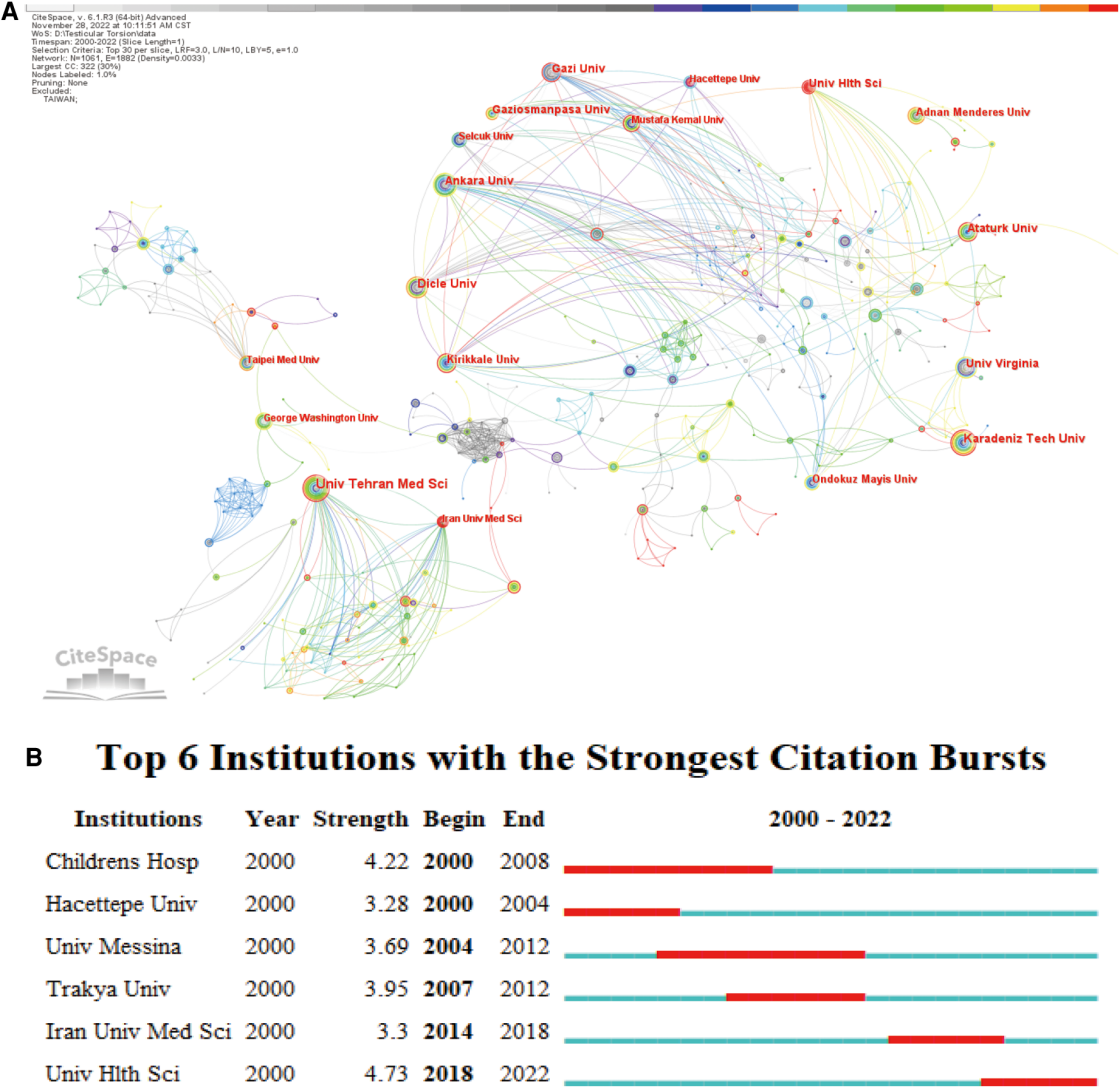
Analysis of institutions involved in research on testicular torsion. (**A**) Network diagram showing institutions links. (**B**) Institutions with periods of burst from 2000 onward among the top 6 burst institutions in articles related to Testicular Torsion.

As the graph shown in Figure 3A, cooperation among institutions was more extensive than that between countries. The most productive institutions, Tehran University Academy of Medical Sciences, worked with Iran Medical University, Urmia University of Medical Sciences, Tehran University of Medical Sciences, University of British Columbia, Tabriz University of Medical Sciences. Among them, Iran University of Medical Sciences and Tehran University of Medical Sciences cooperated closely with Tehran University Academy of Medical Sciences.

Institutional burst analysis found that Boston Children's Hospital, Hacettepe University, University of Messina and other famous foreign institutions had led the research on testicular torsion in the past. In recent years, the influence of Trakya University had gradually increased. The two burst institutions were from Iran and Turkey, which showed that the research on testicular torsion in recent years had been relatively popular in these countries shown in Figure 3B.

#### 3.1.4. Analysis of authors and co-cited authors

**Figure 4:**
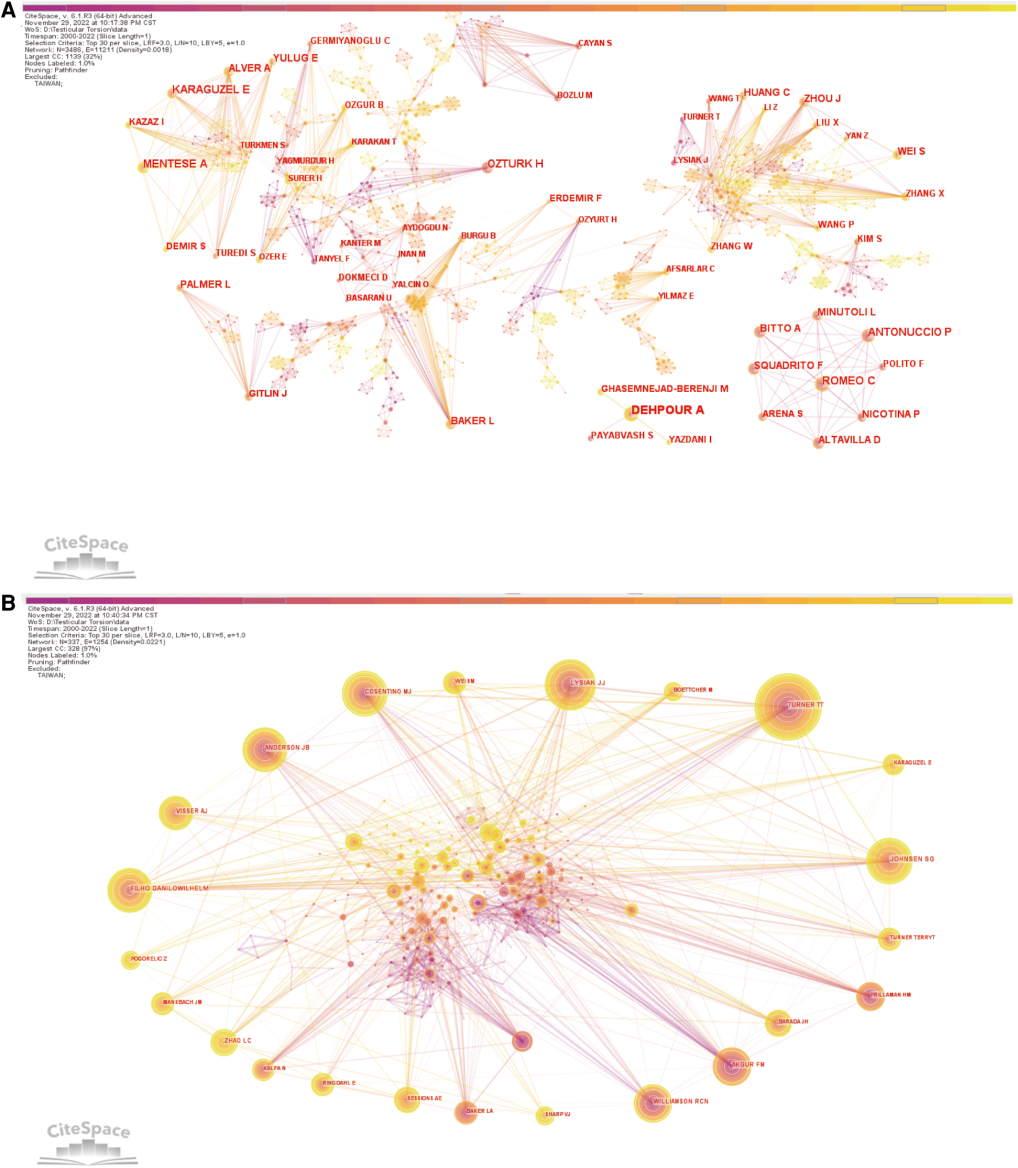
Analysis of authors and co-cited authors involved in research on Testicular Torsion. (**A**) Network diagram showing authors links. (**B**) Network diagram showing co-cited authors links.

More than 3,400 researchers have participated in studies related to testicular torsion. Among them, the three most prolific authors were Ahmad Reza Dehpour (*n* = 16), Carmelo Romeo (*n* = 12) and Ahmet Mentese (*n* = 12). As shown in Table 2, TURNER TT (*n* = 220) ranked 1st among the top 10 co-cited authors, followed by LYSIAK JJ (*n* = 155) and Danilo Wilhelm Filho (*n* = 148). Figure 4 showed the relationship between authors and co-cited authors. A node represented an author, and the size of the node represented the number of published articles. The larger the number of published articles, the larger the node. A few authors cooperated frequently with other authors, such as the Italian author Ahmad Reza Dehpour, who had a larger number and thicker connections with his surrounding authors, which showed that good cooperation may be of great help to obtain more results.

**Table 2:** The top 10 authors and co-cited authors involved in research on Testicular Torsion.

Rank	Author	Count	Co-cited Author	Count
1	DEHPOUR A	16	TURNER TT	220
2	ROMEO C	12	LYSIAK JJ	155
3	MENTESE A	12	FILHO DANILOWILHELM	148
4	KARAGUZEL E	11	JOHNSEN SG	138
5	ANTONUCCIO *P*	10	COSENTINO MJ	124
6	OZTURK H	10	ANDERSON JB	116
7	SQUADRITO F	9	AKGUR FM	105
8	ZHOU J	9	VISSER AJ	98
9	ALTAVILLA D	9	WILLIAMSON RCN	97
10	WEI S	9	ZHAO LC	97

### 3.2. Progress on testicular torsion based on literature analysis

Literature co-citation meant that if two or more articles were cited by one or more papers at the same time, then the two articles were regarded as a co-citation relationship. This relationship can be expressed as the connection between nodes in the graph, which can mine the content of common interest in the literatures and measure the closeness between two articles (16). Citation burst analysis can identify and track publications with strong citation growth characteristics. This surge meant that an article had been quickly cited in the past few years, marking a new turning point in the dynamics of research fields and knowledge (17). We can find the important literature to discover the key content of testicular torsion in the past 20 years.

#### 3.2.1. Analysis of literature co-citation and burst

Among the literatures we retrieved, CiteSpace software was used to the co-citation analysis to generate the top 10 most cited references as shown in Table 3 and the top 30 burst references as shown in Supplementary Figure S1. According to the chart, the figure showed that the first burst of reference began in 2004. The article titled Spermatic cord torsion, reactive oxygen and nitrogen species and ischemia-reperfusion injury concluded: oxidative stress and irreversible oxidative damage caused by ischemia hypoperfusion and ischemia-reperfusion injury caused by prolonged blood supply insufficiency played a major role in testicular torsion (18). And this article was the most cited, and its burst ranked 1st, which showed that in the past 20 years, peer researchers had determined the important position of research on ischemia hypoperfusion and ischemia-reperfusion injury after testicular torsion, and determined the basic direction for future research. The literature titled Management of acute scrotum in children: a 25-year single center experience on 558 pediatric patients ranked 2nd in terms of citations, and also ranked 2nd in burst, which concluded that immediate medical attention and early scrotal exploration in acutely scrotal patients reduced the risk of misdiagnosis of testicular torsion and increased the likelihood of testicular preservation (19). The literature titled Testicular torsion-detorsion and potential therapeutic treatments: A possible role for ischemic postconditioning ranked 3rd in terms of citations, and also ranked 3rd in burst, which described several potential drugs to reduce testicular damage in the ipsilateral and contralateral testis during surgical correction of testicular torsion, but further clinical trials were needed before formal clinical application (20). It was worth noting that nine of the frequently cited articles were published within the past 10 years, indicating that the research results on testicular torsion in recent years have received extensive attention from peer researchers and may still be at the core of future research.

**Table 3:** Ranking of the number of the cited references.

Rank	Count	Year	Cited references
1	32	2004	Filho DANILOWILHELM, 2004, MOL ASPECTS MED, V25, P199, DOI 10.1016/j.mam.2004.02.020
2	30	2016	Pogorelic Z, 2016, CAN J UROL, V23, P8594
3	30	2016	Shimizu S, 2016, INT J UROL, V23, P454, DOI 10.1111/iju.13110
4	23	2019	Mellick LB, 2019, PEDIATR EMERG CARE, V35, P821, DOI 10.1097/PEC.0000000000001287
5	22	2011	Zhao LC, 2011, J UROLOGY, V186, P2009, DOI 10.1016/j.juro.2011.07.024
6	22	2014	Karaguzel E, 2014, NAT REV UROL, V11, P391, DOI 10.1038/nrurol.2014.135
7	21	2016	Sheth KR, 2016, J UROLOGY, V195, P1870, DOI 10.1016/j.juro.2016.01.101
8	20	2018	Bandarkar AN, 2018, PEDIATR RADIOL, V48, P735, DOI 10.1007/s00247-018-4093-0
9	19	2017	Arena S, 2017, EXP THER MED, V13, P2115, DOI 10.3892/etm.2017.4289
10	19	2020	Jacobsen FM, 2020, WORLD J MENS HEALTH, V38, P298, DOI 10.5534/wjmh.190037

From the burst time of these 30 publications, the research on testicular torsion ischemia-reperfusion injury in the past 20 years had carried out the whole course of testicular torsion study. Among them, a total of 11 publications were biased toward basic research, which focused on establishing rat model of testicular torsion and further exploring the mechanisms by which testicular torsion-de-torsion (ischemia-reperfusion) injury triggered the production of reactive oxygen species (ROS), proinflammatory cytokines, neutrophil recruitment, lipid peroxidation, hypoxia, and apoptosis and the mechanisms by which various drug applications aggravated or mitigated testicular torsion (20).

The literature on basic research 10 years ago primarily described factors aggravating testicular torsion, such as 1) activation of TNF*α* and IL-1β and JNK pathways with e-selectin expression and neutrophil recruitment to the testis involved in testicular ischemia-reperfusion (21); 2) elevated NO levels likely promoted peroxynitrite production involved in post-ischemic perfusion deficit and ischemia-reperfusion injury in testicular torsion (18); 3) reactive oxygen species (ROS), activated mitogen-activated protein kinases (MAPKs), PPAR *β*/*δ* receptors, induced transcription factors and growth factors including NF- *κ* B and VE GF were closely associated with damage triggered by the ischemia reperfusion program (22). In contrast, the literature on basic research in the past 10 years had focused on factors protecting against testicular torsion. For example, 1) administration of nifedipine before torsion decreased ipsilateral testicular malondialdehyde levels indirectly elevating superoxide dismutase and glutathione peroxidase activities that prevented testicular tissue ischemia/reperfusion cell injury (23); 2) administration of melatonin resulted in a significant decrease in ipsilateral testicular lipid peroxidation and enzyme activity favoring protection against testicular torsion (24); 3) N-acetylcysteine, hydroalcoholic extract of Fumaria minor, and aminophene sulfone administered during ischemia/reperfusion (I-R) counteracted testicular torsion (25–27); 4) Application IPostC may inhibit the expression of mitogen-activated protein kinase, inflammatory cytokines (IL-6 and TNF-a) and NF-KB, enhanced the expression of IKB-a and reduced histological damage to the testis by I-R, and may also reduce tissue ischemia by reducing the sudden production of ROS during ischemia-reperfusion through gradual reperfusion (20). The above content showed that the basic research in the past 10 years preferred the protection of ischemia-reperfusion injury and developed drugs or chemicals for the clinical treatment of testicular torsion. In addition, a total of 19 burst publications were biased towards clinical studies, and almost all of the literature emphasized the importance of early diagnosis, early treatment, and early intervention for testicular torsion (6, 7, 28, 29)., etc. An article published in 2003 highlighted the role of education in the general population in the early detection of testicular torsion and, more importantly, in improving its prognosis (28). Two articles had emphasized that early rigorous physical examination can reduce the risk of misdiagnosis of testicular torsion (6, 30). Meanwhile, relevant literature pointed out that color ultrasound examination indicated the existence of intravessticular blood flow, and manual reduction of reversed testes was still needed (31). Sheth KR et al. established the The TWIST (Testicular Workup for Ischemia and Suspected Torsion) score, with parameters including testicular swelling, hard testis, no testicular reflex, nausea/vomiting and high riding testis, indicating that high-risk patients should undergo immediate surgical exploration and further imaging studies should not be performed (7, 8). In general, in the past 20 years, the literature on testicular torsion had shown the phenomenon of common progress between basic experiments and clinical research. Scientists in this field had jointly participated in the study of the early detection, early diagnosis, early treatment, and the protection of ischemia and reperfusion after testicular torsion. In general, in the past 20 years, the literature on testicular torsion had shown the phenomenon of common progress between basic experiments and clinical research. Scientists in this field have jointly participated in the study of the early detection, early diagnosis, early treatment, and the protection of ischemia and reperfusion after testicular torsion.

### 3.3. Progress on testicular torsion based on keyword analysis

Keywords were words or phrases that reflected the characteristics of a paper. Based on the CiteSpace software, the keyword co-citation analysis can be used to understand the research hotspots and emerging trends of testicular torsion, as well as the keyword burst analysis to understand the frontier fields (32). In this study, we used the CiteSpace software to generate the keyword map of the testicular torsion research, and further explored the historical evolution of the hot spots in this field.

#### 3.3.1. Analysis of keyword frequency

Keyword visualization analysis of the literature on testicular torsion from January 2000-November 2022 was performed using CiteSpace software, the parameters were set as follows: 1. time slice was 1 year, 2. each slice selected top30 keywords, 3. merged similar keywords such as testicular torsion, spermatic cord torsion, testicular torsion-detorsion, ischemia reperfusion injury, ischemia/reperfusion, ischemia-reperfusion injury etc. Finally, the Pathfinder Network method was used to obtain Table 1. As the Supplementary Table S1 showed, the top 30 hot keywords word frequency was more than 25 times. The most frequent keyword was “testicular torsion”, with a frequency of 700 times, followed by “ischemia reperfusion injury”, with a frequency of 235 times. Other keywords with high frequency included “acute scrotum”, “children”, “oxidative stress”, etc., which suggested that “testicular torsion”, “children”, “diagnosis”, “color doppler sonography”, “orchiectomy” were the most important components for the development of acute scrotal studies, “ischemia reperfusion injury”, “oxidative stress “ischemia reperfusion injury”, “oxidative stress”, and “blood flow” and “spermatogenesis” were very important mechanisms to study in testicular torsion.

#### 3.3.2. Analysis of keyword cluster and burst

**Figure 5:**
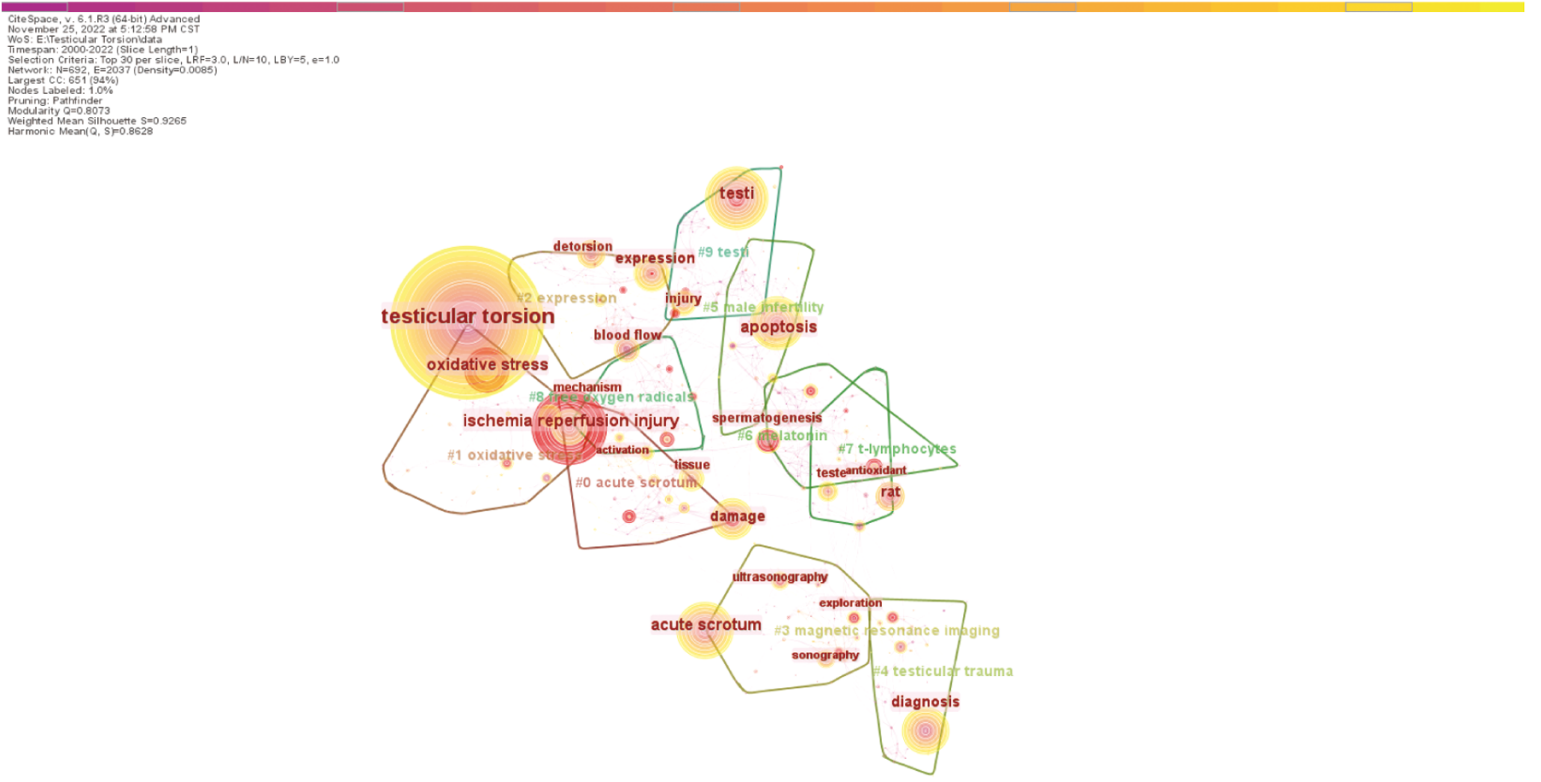
Analysis of keyword cluster map in testicular torsion study. (Timespan: 2000–2022; Slice Length: 1; Selection Criteria: Top 30 per slice; Network: N = 692, E = 2037; Density: 0.0085; Largest CC:651; Nodes Labeled:1.0%; Pruning: Pathfinder; Modularity Q:0.8073; Weighted Mean Silhouette S:0.9265).

The effect of the graph drawn by CiteSpace software can be measured by Q value and S value. Q-value referred to Modularity, which generally considered that the cluster structure generated is significant with Q > 0.3. And the S-value referred to Silhouette, and clustering is generally considered to be reasonable with S > 0.5, S > 0.7 indicated that clustering is efficient and convincing. The visualization map analysis yielded 9 major keyword clusters, as shown in Figure 5, with *Q* value = 0.8073, indicating significant clustering structure and good clustering effect. The value of S = 0.9265, which was not only much larger than the critical value of 0.5, but also larger than 0.7, indicating that the clustering results were convincing. The high-quality cluster results were helpful to further analysis the general characteristics and development trend of testicular torsion. The main cluster table of the top 14 keywords related to testicular torsion was drawn by keyword cluster analysis and showed as Supplementary Table S2, and the results we obtained were also convincing with S > 0.7. The size of the first 14 clusters is all over 30. The maximum cluster had 53 members, labelled as acute scrotum, and its main keyword was acute scrotum; ischemia-reperfusion injury; testicular torsion; detorsion; tissue; diagnosis, which also explained the importance of testicular torsion in acute testis. The remaining label clusters were successively “oxidative stress” (*n* = 47), “expression” (*n* = 45), “acute scrotum” (*n* = 42), “testicular trauma” (*n* = 41), “male infertility” (*n* = 40) and so on.

**Figure 6:**
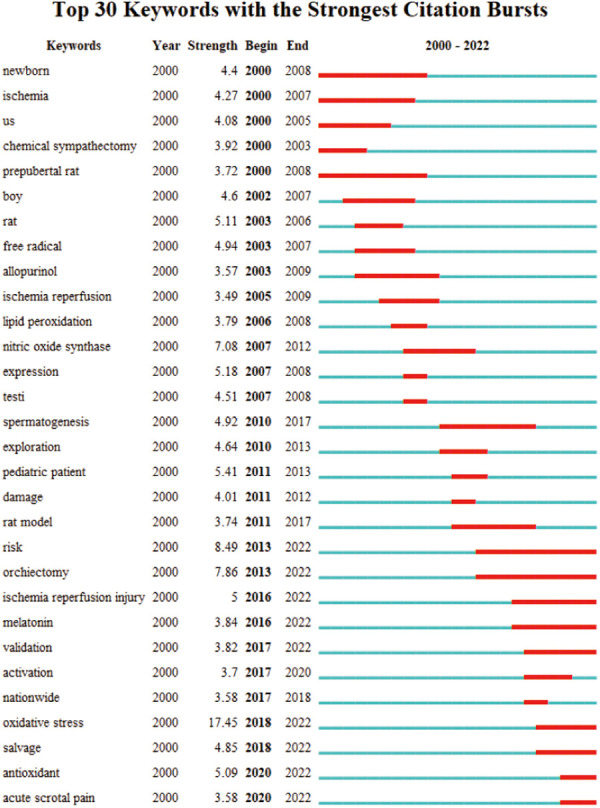
The top 30 keywords sorted by the beginning year of burst.

Color doppler ultrasound was mentioned in clusters #3 and #4 indicating its indispensable auxiliary examination in clinical application and its importance in early detection of testicular torsion. The option of surgical exploration and even unilateral orchidectomy in patients with testicular torsion were mentioned repeatedly in #3, #12, and #15, which again argued that the necessity of surgery when patients were considered to testicular torsion, even when auxiliary examination was helpful to rule out the possibility of testicular torsion (7). In addition, we found that all of these 14 clusters appeared during the 2000–2010 period, illustrating that research on testicular torsion is more advanced within the last decade than it was 10 years ago. Therefore, peer researchers were needed to conduct in-depth analysis of the causes, and to initiate new studies on testicular torsion to help clinicians in their medical work. According to keyword co-citation analysis, CiteSpace software was used to generate the top 30 keywords with the strongest citation burst as shown in Figure 6. We found that the strongest burst keyword was “oxidative stress”. Similar to other organs, torsion damage of testis was closely related to oxidative stress, and the free radical oxidation of free lipids and cholesterol in cell membrane and lipoprotein is called lipid oxidation, which played an important role in ischemia-reperfusion. The keyword “oxidative stress” had burst from 2018 to the present and will continue to play a vital keyword role in the future (18). “Orchiectomy” was the second ranked keyword for burst. Considering the consultation time and misdiagnosis of patients with testicular torsion, which led to a relatively high selectivity of about 42% for orchiectomy in scrotal exploration, this might be one of the reasons for the popularity of this keyword (7, 10, 33).

The burst time of “prepubertal”, “rat” and “rat model” lasted throughout the 20 years, meant that basic research on testicular torsion is very close to rats, which showed that the researcher was good at establishing the rat testicular torsion model to monitor the changes of “nitric oxide synthase”, “antioxidant” and “allopurinol” and so on in testicle ischemia-reperfusion to estimate their potential clinical value.

## 4. Discussion

In this study, we searched for articles on testicular torsion between 2000 and 2022, all of which were obtained from the Web of Science database. After our selection, we got 1,007 English literatures that had been published all over the world. Subsequently, CiteSpace was used to perform analysis on the basic indicators and burst keywords in the literature in this field, so as to achieve quantitative and visual evaluation. The results of a bibliometric analysis of testicular torsion over the past 20 years were as follows. In summary, the number of articles published in this field was on an increasing trend, demonstrating that the research on testicular torsion was gradually getting attention. However, attention had not been particularly high, which may be related to the low incidence of testicular torsion (34). According to the analysis of countries and regions, the top 10 countries and regions published articles accounting for 79.05% of the total published articles, indicating that these countries and regions participating in the research in this field were mainly concentrated in a few countries, while other countries and regions had little participation. The United States, Turkey, China were the major countries in the study of testicular torsion. They each had their own cooperative countries and regions, but there was a lack of cooperation among these major countries. The institutional analysis found that institutions from Turkey ranked highly in both publication production and burst analysis, indicating the continuing popularity of testicular torsion research in Turkey.

And most institutions were mainly concentrated in universities, while a few were concentrated in hospitals. Many countries and multiple institutions should strengthen exchanges and cooperation, and different regions and different disciplines should also exchange and share the research results of testicular torsion to promote the continuous progress of this study. The most prolific author was Ahmad Reza Dehpour researcher from Italy, while TURNER TT was the most cited American author, both of whom had made outstanding contributions to the development of testicular torsion. The author Ahmad Reza Dehpour focused mainly on the study of ischemia-reperfusion after testicular torsion in a rat model. And he showed that metformin may play a major role in the cytoprotective effect of reducing oxidative stress and apoptosis by establishing experiments in a rat model of testicular torsion (35). Moreover, he also found that modafinil reduces testicular ischemia-reperfusion injury to have a protective effect against testicular torsion in rats. In addition, the author Turner TT focused on molecular apoptotic pathways in germ cells after acute ischemia in the rat testis. He found that germ cell apoptosis after testicular ischemia/reperfusion in rats is triggered by mitochondria-associated molecules Bax as well as Fas-FasL interactions (36). And he revealed that the mitochondrial cystatin-9-dependent pathway mediates testicular torsion germ cell specific apoptosis, providing a broader context for the development of therapeutic interventions to rescue testicular function (37).

Through the co-citation analysis of the literature, we found that the theme of the burst literature in the past 20 years was still the early detection, diagnosis and treatment of testicular torsion, and the ischemia-reperfusion injury after testicular torsion was still the focus of basic researchers. On the one hand, clinicians had developed testicular torsion scores to assist in the differential diagnosis of acute testicular patients (38). They also calculated the formula derived from the duration of symptoms and the degree of twisting to determine the viability of the torsion testis, to preliminarily judge the testicular salvage rate of testicular torsion (5). The literature also suggested improving the prognosis of testicular torsion by educating the general population, which we also believe is a viable approach (28). On the other hand, basic researchers had focused on exploring the pathophysiological mechanisms of ischemia-reperfusion injury after testicular torsion and developing drugs or chemicals related to the aggravation or mitigation of testicular torsion. Although related drugs or chemicals can improve the prognosis of testicular torsion, they were limited to rat model of testicular torsion and were still a long way from real clinical application, and more research was needed for clinical translation in the future. Keyword analysis allowed us to identify research priorities in the field over the past 20 years, the most frequent keywords related to testicular torsion were “testicular torsion” and “ischemia reperfusion injury”, the main cluster labels were “acute scrotum” and “oxidative stress”, and the keywords with the strongest burst were “oxidative stress”, “risk”. These indicated that the hotspots of research on testicular torsion were clustered in the direction of ischemia-reperfusion injury and oxidative stress, and we believed that these aspects will remain central in future research. The current “gold standard” examination and treatment for testicular torsion was an emergency scrotal exploration to untwist the testicle (39, 40). However, findings during scrotal exploration might vary and might be ambiguous, meaning that the surgeon needs to make a series of potentially complex intraoperative decisions (41). There was no high-quality evidence on scrotal exploration decision-making and conduct. A systematic evaluation included 182 studies and 3 patients detailing different surgical techniques, highlighting the lack of strong evidence to support any specific approach for short-term complications or long-term outcomes such as reversal, fertility, or patient-reported outcomes (42). Furthermore, any specific surgical technique or decision-making process was not currently recommended according to the EAU (The European Association of Urology) Guidelines on Paediatric Urology (1). However, timely recognition and treatment were necessary for testicular salvage, and torsion must be excluded in all acute scrotal patients (7). In addition, considering the low age of onset, the high rate of misdiagnosis and the serious adverse consequences of damaging spermatogenesis, more research needed to be devoted to testicular torsion to improve the success rate of testicular salvage (7, 11, 34). To our knowledge, this study was the first bibliometric analysis focusing on research on testicular torsion. This article explained the historical evolution of the field and explored its hotspot areas by downloading the WoSCC database literature and constructing multiple knowledge maps of testicular torsion using CiteSpace.

However, there were some limitations regarding this study. First, we only downloaded and analyzed English articles indexed in the Web of Science database, excluding non-English articles, other types of literature, and relevant articles not indexed in the WoSCC database. Therefore, some artificial biases may lead to incomplete results. More databases were needed in the future, and even some non-English literature can be introduced to participate in the analysis. The problem of information overload made it difficult for people to obtain valuable information. Bibliometrics is a mature literature analysis and information mining method, which has obvious objectivity and advantages in quantitative and modeling macro research. It takes the external characteristics of scientific literature as the research object, and studies the distribution structure, quantitative relationship and changing law of the literature (43). Compared with traditional reviews, bibliometric research can investigate more data to maintain a high degree of rigor, scientific soundness, transparency and replicability (44, 45). CiteSpace, as a tool for bibliometric analysis, could be used frequently for many previous data visualization and analysis work by downloading relevant literature from specific databases (46). In addition, it could quickly discover the research trajectory, current hot research and frontiers of a field, which would help researchers to quickly and comprehensively understand the development of the field and determine the research direction (47, 48). Therefore, we still believed that this study can be used to describe the general situation and trends in this field from 2000 to 2022, and to provide ideas for subsequent in-depth studies.

## 5. Conclusion

Our results suggested that since 2000, a large number of high-quality literature on testicular torsion had been published, and the depth and breadth of research were gradually expanded. With the help of information visualization through analysis by CiteSpace software, we had identified research hotspots and priorities in the field, and predicted research trends and provided reference information for future researchers. We believed that the number of countries and regions involved in this research will continue to increase, and there will be closer cooperation between countries and institutions. The research forms of basic experiments and clinical research will continue to diversify. The research topic continued to focus on the early diagnosis and treatment of testicular torsion. In addition, protection of ischemia-reperfusion after testicular torsion will continue to be explored.

## Data Availability

The original contributions presented in the study are included in the article/**Supplementary Material**, further inquiries can be directed to the corresponding author.

## References

[1] Keays M Rosenberg H. Testicular torsion. CMAJ. (2019) 191(28):E792. 10.1503/cmaj.190158 31308008 PMC6629539

[2] Dang VT Pradere B de Varennes AM Benali NA Vallee M Berchiche W Torsion of the spermatic cord in adults: a multicenter experience in adults with surgical exploration for acute scrotal pain with suspected testicular torsion. Asian J Androl. (2022) 24(6):575–8. 10.4103/aja2021126 35322657 PMC9809486

[3] Qin KR Qu LG. Diagnosing with a TWIST: systematic review and meta-analysis of a testicular torsion risk score. J Urol. (2022) 208(1):62–70. 10.1097/JU.0000000000002496 35238603

[4] Deeg KH. Differential diagnosis of acute scrotum in childhood and adolescence with high-resolution Duplex sonography. Ultraschall Med. (2021) 42(1):10–38. 10.1055/a-1325-1834 33530122

[5] Howe AS Vasudevan V Kongnyuy M Rychik K Thomas LA Matuskova M Degree of twisting and duration of symptoms are prognostic factors of testis salvage during episodes of testicular torsion. Transl Androl Urol. (2017) 6(6):1159–66. 10.21037/tau.2017.09.10 29354505 PMC5760391

[6] Mestrovic J Pogorelic Z Drmic-Hofman I Vilovic K Todoric D Popovic M. Protective effect of urapidil on testicular torsion-detorsion injury in rats. Surg Today. (2017) 47(3):393–8. 10.1007/s00595-016-1388-3 27444029

[7] Sharp VJ Kieran K Arlen AM. Testicular torsion: diagnosis, evaluation, and management. Am Fam Physician. (2013) 88(12):835–40. 10.1136/bmj.f7437 24364548

[8] Lian BS Ong CC Chiang LW Rai R Nah SA. Factors predicting testicular atrophy after testicular salvage following torsion. Eur J Pediatr Surg. (2016) 26(1):17–21. 10.1055/s-0035-1566096 26509312

[9] Strobel MC Seperack PK Copeland NG Jenkins NA. Molecular analysis of two mouse dilute locus deletion mutations: spontaneous dilute lethal20J and radiation-induced dilute prenatal lethal Aa2 alleles. Mol Cell Biol. (1990) 10(2):501–9. 10.1128/MCB.10.2.501 2300051 PMC360824

[10] Cost NG Bush NC Barber TD Huang R Baker LA. Pediatric testicular torsion: demographics of national orchiopexy versus orchiectomy rates. J Urol. (2011) 185(6 Suppl):2459–63. 10.1016/j.juro.2011.01.016 21527194

[11] Jacobsen FM Rudlang TM Fode M Ostergren PB Sonksen J Ohl DA The impact of testicular torsion on testicular function. World J Mens Health. (2020) 38(3):298–307. 10.5534/wjmh.190037 31081295 PMC7308234

[12] Ta A D'Arcy FT Hoag N D'Arcy JP Lawrentschuk N. Testicular torsion and the acute scrotum: current emergency management. Eur J Emerg Med. (2016) 23(3):160–5. 10.1097/MEJ.0000000000000303 26267075

[13] Arena S Iacona R Antonuccio P Russo T Salvo V Gitto E Medical perspective in testicular ischemia-reperfusion injury. Exp Ther Med. (2017) 13(5):2115–22. 10.3892/etm.2017.4289 28565817 PMC5443302

[14] Tan Z Dong Y Li Q. Dynamics of acute postsurgical pain over the last decade: a bibliometric analysis. Pain Res Manag. (2022) 2022:8090209. 10.1155/2022/8090209 36385903 PMC9663218

[15] Zhu X Hu J Deng S Tan Y Qiu C Zhang M Bibliometric and visual analysis of research on the links between the gut Microbiota and depression from 1999 to 2019. Front Psychiatry. (2020) 11:587670. 10.3389/fpsyt.2020.587670 33488420 PMC7819979

[16] Yan SJ Chen M Wen J Fu WN Song XY Chen HJ Global research trends in cardiac arrest research: a visual analysis of the literature based on CiteSpace. World J Emerg Med. (2022) 13(4):290–6. 10.5847/wjem.j.1920-8642.2022.071 35837560 PMC9233974

[17] Li Z Shang W Wang C Yang K Guo J. Characteristics and trends in acceptance and commitment therapy research: a bibliometric analysis. Front Psychol. (2022) 13:980848. 10.3389/fpsyg.2022.980848 36452380 PMC9702511

[18] Filho DW Torres MA Bordin AL Crezcynski-Pasa TB Boveris A. Spermatic cord torsion, reactive oxygen and nitrogen species and ischemia-reperfusion injury. Mol Aspects Med. (2004) 25(1-2):199–210. 10.1016/j.mam.2004.02.020 15051328

[19] Pogorelic Z Mustapic K Jukic M Todoric J Mrklic I Messtrovic J Management of acute scrotum in children: a 25-year single center experience on 558 pediatric patients. Can J Urol. (2016) 23(6):8594–601. 27995859

[20] Shimizu S Tsounapi P Dimitriadis F Higashi Y Shimizu T Saito M. Testicular torsion-detorsion and potential therapeutic treatments: a possible role for ischemic postconditioning. Int J Urol. (2016) 23(6):454–63. 10.1111/iju.13110 27217335

[21] Lysiak JJ Nguyen QA Kirby JL Turner TT. Ischemia-reperfusion of the murine testis stimulates the expression of proinflammatory cytokines and activation of c-jun N-terminal kinase in a pathway to E-selectin expression. Biol Reprod. (2003) 69(1):202–10. 10.1095/biolreprod.102.013318 12620934

[22] Karaguzel E Kadihasanoglu M Kutlu O. Mechanisms of testicular torsion and potential protective agents. Nat Rev Urol. (2014) 11(7):391–9. 10.1038/nrurol.2014.135 24934447

[23] Mestrovic J Drmic-Hofman I Pogorelic Z Vilovic K Supe-Domic D Seselja-Perisin A Beneficial effect of nifedipine on testicular torsion-detorsion injury in rats. Urology. (2014) 84(5):1194–8. 10.1016/j.urology.2014.07.022 25443933

[24] Parlaktas BS Atilgan D Ozyurt H Gencten Y Akbas A Erdemir F The biochemical effects of ischemia-reperfusion injury in the ipsilateral and contralateral testes of rats and the protective role of melatonin. Asian J Androl. (2014) 16(2):314–8. 10.4103/1008-682X.122202 24407181 PMC3955347

[25] Aktas BK Bulut S Bulut S Baykam MM Ozden C Senes M The effects of N-acetylcysteine on testicular damage in experimental testicular ischemia/reperfusion injury. Pediatr Surg Int. (2010) 26(3):293–8. 10.1007/s00383-009-2538-0 19911182

[26] Shokoohi M Shoorei H Soltani M Abtahi-Eivari SH Salimnejad R Moghimian M. Protective effects of the hydroalcoholic extract of fumaria parviflora on testicular injury induced by torsion/detorsion in adult rats. Andrologia. (2018) 50(7):e13047. 10.1111/and.13047 29770471

[27] Dejban P Rahimi N Takzare N Jahansouz M Haddadi NS Dehpour AR. Beneficial effects of dapsone on ischemia/reperfusion injury following torsion/detorsion in ipsilateral and contralateral testes in rat. Theriogenology. (2019) 140:136–42. 10.1016/j.theriogenology.2019.08.021 31473496

[28] Visser AJ Heyns CF. Testicular function after torsion of the spermatic cord. BJU Int. (2003) 92(3):200–3. 10.1046/j.1464-410X.2003.04307.x 12887467

[29] Ringdahl E Teague L. Testicular torsion. Am Fam Physician. (2006) 74(10):1739–43. 10.1016/j.pop.2006.09.008 17137004

[30] Bayne CE Villanueva J Davis TD Pohl HG Rushton HG. Factors associated with delayed presentation and misdiagnosis of testicular torsion: a case-control study. J Pediatr. (2017) 186:200–4. 10.1016/j.jpeds.2017.03.037 28427778

[31] Bandarkar AN Blask AR. Testicular torsion with preserved flow: key sonographic features and value-added approach to diagnosis. Pediatr Radiol. (2018) 48(5):735–44. 10.1007/s00247-018-4093-0 29468365 PMC5895684

[32] Li Y Gao Q Chen N Zhang Y Wang J Li C Clinical studies of magnetic resonance elastography from 1995 to 2021: scientometric and visualization analysis based on CiteSpace. Quant Imaging Med Surg. (2022) 12(11):5080–100. 10.21037/qims-22-207 36330182 PMC9622435

[33] Zhao LC Lautz TB Meeks JJ Maizels M. Pediatric testicular torsion epidemiology using a national database: incidence, risk of orchiectomy and possible measures toward improving the quality of care. J Urol. (2011) 186(5):2009–13. 10.1016/j.juro.2011.07.024 21944120

[34] Brown TW McCarthy ML Kelen GD Levy F. An epidemiologic study of closed emergency department malpractice claims in a national database of physician malpractice insurers. Acad Emerg Med. (2010) 17(5):553–60. 10.1111/j.1553-2712.2010.00729.x 20536812

[35] Ghasemnejad-Berenji M Ghazi-Khansari M Yazdani I Nobakht M Abdollahi A Ghasemnejad-Berenji H Effect of metformin on germ cell-specific apoptosis, oxidative stress and epididymal sperm quality after testicular torsion/detorsion in rats. Andrologia. (2018) 50(2):e12846. 10.1111/and.12846 28730645

[36] Lysiak JJ Turner SD Turner TT. Molecular pathway of germ cell apoptosis following ischemia/reperfusion of the rat testis. Biol Reprod. (2000) 63(5):1465–72. 10.1095/biolreprod63.5.1465 11058553

[37] Lysiak JJ Zheng S Woodson R Turner TT. Caspase-9-dependent pathway to murine germ cell apoptosis: mediation by oxidative stress, BAX, and caspase 2. Cell Tissue Res. (2007) 328(2):411–9. 10.1007/s00441-006-0341-y 17265069

[38] Sheth KR Keays M Grimsby GM Granberg CF Menon VS DaJusta DG Diagnosing testicular torsion before urological consultation and imaging: validation of the TWIST score. J Urol. (2016) 195(6):1870–6. 10.1016/j.juro.2016.01.101 26835833

[39] Mellick LB Sinex JE Gibson RW Mears K. A systematic review of testicle survival time after a torsion event. Pediatr Emerg Care. (2019) 35(12):821–5. 10.1097/PEC.0000000000001287 28953100

[40] DaJusta DG Granberg CF Villanueva C Baker LA. Contemporary review of testicular torsion: new concepts, emerging technologies and potential therapeutics. J Pediatr Urol. (2013) 9(6 Pt A):723–30. 10.1016/j.jpurol.2012.08.012 23044376 PMC3566290

[41] Clement KD Light A Asif A Chan VW Khadhouri S Shah TT A BURST-BAUS consensus document for best practice in the conduct of scrotal exploration for suspected testicular torsion: the finding consensus for orchIdopeXy in torsion (FIX-IT) study. BJU Int. (2022) 130(5):662–70. 10.1111/bju.15818 35689399 PMC9796508

[42] Moore SL Chebbout R Cumberbatch M Bondad J Forster L Hendry J Orchidopexy for testicular torsion: a systematic review of surgical technique. Eur Urol Focus. (2021) 7(6):1493–503. 10.1016/j.euf.2020.07.006 32863201

[43] Bornmann L. and Marx W, Critical rationalism and the search for standard (field-normalized) indicators in bibliometrics. J. Informetr. (2018) 12(3): p. 598–604. 10.1016/j.joi.2018.05.002

[44] Dada O., A model of entrepreneurial autonomy in franchised outlets: a systematic review of the empirical evidence. Int. J. Manag. Rev. (2016) 20(2):206–26. 10.1111/ijmr.12123

[45] Rey-Marti A Ribeiro-Soriano D. Palacios-Marques. A Bibliometric Analysis of Social Entrepreneurship. (2016) 69(5):1651–5. 10.1016/j.jbusres.2015.10.033

[46] Yang W Wang S Chen C Leung HH Zeng Q Su X. Knowledge mapping of enterprise network research in China: a visual analysis using CiteSpace. Front Psychol. (2022) 13:898538. 10.3389/fpsyg.2022.898538 35846692 PMC9282046

[47] Chen YH Yin MQ Fan LH Jiang XC Xu HF Zhang T Bibliometric analysis of traditional Chinese medicine research on heart failure in the 21st century based on the WOS database. Heliyon. (2023) 9(1):e12770. 10.1016/j.heliyon.2022.e12770 36691539 PMC9860440

[48] Zhong D Li Y Huang Y Hong X Li J Jin R. Molecular mechanisms of exercise on cancer: a bibliometrics study and visualization analysis via CiteSpace. Front Mol Biosci. (2021) 8:797902. 10.3389/fmolb.2021.797902 35096970 PMC8794585

