# Supplementary material for: Mapping trends and hotspot regarding testicular torsion: A bibliometric analysis of global research (2000–2022)
Source: Front Pediatr. 2023 Feb 28;11:1121677. doi: 10.3389/fped.2023.1121677 (PMC10011162; doi:10.3389/fped.2023.1121677)
Supplement: Supplementary file 1 [file Datasheet1.pdf]

Strategies for literature search in testicular torsion:

Databases: SCI-EXPANDED, CPCI-S, CPCI-SSH, BKCI-S, BKCI-SSH

#1: TI=(“spermatic cord torsion*”) OR TI=(“twisting of the spermatic cord”) OR TI=(“torsion of testicular cord”) OR TI=(“testicular torsion*”) OR TI=(“pediatric testicular torsion”)
#2: AB=(“spermatic cord torsion*”) OR AB=(“twisting of the spermatic cord”) OR AB=(“torsion of testicular cord”) OR AB=(“testicular torsion*”) OR AB=(“pediatric testicular torsion”)
((#1 OR #2) AND DT=(Article)) AND LA=(English)
DOP: 2000-01-01 to 2022-11-01

Table S1 Ranking of top 30 high-frequency keyword in the testicular torsion study

Rank	Count	Year	Keyword	Rank	Count	Year	Keyword
1	700	2000	testicular torsion	16	47	2000	contralateral testi
2	235	2000	ischemia reperfusion injury	17	46	2000	nitric oxide
3	169	2000	testi	18	45	2000	color doppler sonography
4	157	2000	children	19	45	2000	injury
5	156	2001	apoptosis	20	45	2000	spermatogenesis
6	133	2000	acute scrotum	21	44	2000	blood flow
7	129	2004	oxidative stress	22	44	2000	ischemia
8	101	2000	diagnosis	23	41	2013	risk
9	88	2000	management	24	38	2001	mechanism
10	86	2001	damage	25	38	2013	orchiectomy
11	83	2001	expression	26	37	2003	teste
12	61	2001	rat	27	34	2000	ultrasonography
13	55	2000	ultrasound	28	33	2000	sonography
14	50	2000	detorsion	29	30	2004	antioxidant
15	49	2001	tissue	30	26	2005	exploration

Table S2 Ranking of top 14 keywords main clusters in testicular torsion from 2000 to 2022

Cluster ID	Size	Sihouette	Mean year	Label（LLR, log-likelihood ratio）
#0	53	0.913	2007	acute scrotum; ischemia-reperfusion injury; testicular torsion detorsion; tissue; diagnosis
#1	47	0.958	2006	oxidative stress; apoptosis; children; management; tunica albuginea
#2	45	0.867	2007	expression; cell survival; acute scrotum; nitric oxide synthase; adhesion molecules

#3	42	0.891	2005	acute scrotum; magnetic resonance imaging; oxidative stress; sonography; exploration
#4	41	0.91	2005	testicular trauma; diagnosis; children; spermatic cord; color doppler sonography
#5	40	0.95	2005	male infertility; pioglitazone; testicular ischaemia; testes torsion; antioxidant
#6	39	0.936	2002	melatonin; teste; testicular cancer; hcg stimulation; acid
#7	37	0.941	2005	t-lymphocytes; testicular tumor; ejaculation; purinergic transmission; tnf-alpha/tnfr1
#8	35	0.88	2006	free oxygen radicals; epidermal growth factor; male reproductive tract; garlic extract; sphingosine-1-phosphate
#9	34	0.928	2003	testi; climate; erythropoietin; testicular ischemia-reperfusion; basal membrane
#10	34	0.964	2004	undescended testis; testis-undescended; testis-torsion; minimally invasive surgery; testis-nonpalpable
#11	33	0.976	2006	acute scrotal pain; management; newborn; apoptosis; oxidative stress
#12	32	0.876	2007	reperfusion injury; nitric oxide; contralateral testis; sildenafil; acute scrotum
#13	32	0.924	2006	cryptorchidism; delay; pediatric urology; orchiectomy; orchiopexy

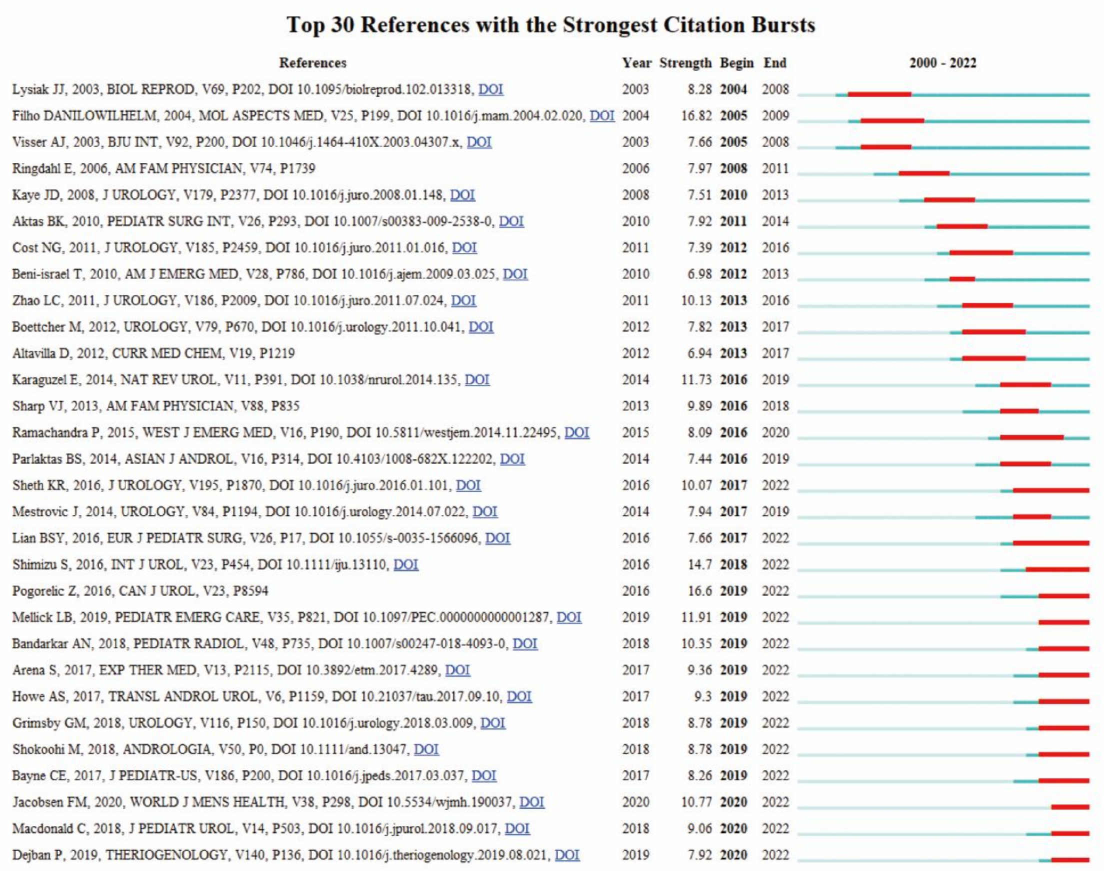

Top 30 References with the Strongest Citation Bursts

References	Year	Strength	Begin	End	2000 - 2022
Lysiak JJ, 2003, BIOL REPROD, V69, P202, DOI 10.1095/biolreprod.102.013318, DOI	2003	8.28	**2004**	2008	
Filho DANILOWILHELM, 2004, MOL ASPECTS MED, V25, P199, DOI 10.1016/j.mam.2004.02.020, DOI	2004	16.82	**2005**	2009	
Visser AJ, 2003, BJU INT, V92, P200, DOI 10.1046/j.1464-410X.2003.04307.x, DOI	2003	7.66	**2005**	2008	
Ringdahl E, 2006, AM FAM PHYSICIAN, V74, P1739	2006	7.97	**2008**	2011	
Kaye JD, 2008, J UROLOGY, V179, P2377, DOI 10.1016/j.juro.2008.01.148, DOI	2008	7.51	**2010**	2013	
Aktas BK, 2010, PEDIATR SURG INT, V26, P293, DOI 10.1007/s00383-009-2538-0, DOI	2010	7.92	**2011**	2014	
Cost NG, 2011, J UROLOGY, V185, P2459, DOI 10.1016/j.juro.2011.01.016, DOI	2011	7.39	**2012**	2016	
Beni-israel T, 2010, AM J EMERG MED, V28, P786, DOI 10.1016/j.ajem.2009.03.025, DOI	2010	6.98	**2012**	2013	
Zhao LC, 2011, J UROLOGY, V186, P2009, DOI 10.1016/j.juro.2011.07.024, DOI	2011	10.13	**2013**	2016	
Boettcher M, 2012, UROLOGY, V79, P670, DOI 10.1016/j.urology.2011.10.041, DOI	2012	7.82	**2013**	2017	
Altavilla D, 2012, CURR MED CHEM, V19, P1219	2012	6.94	**2013**	2017	
Karaguzel E, 2014, NAT REV UROL, V11, P391, DOI 10.1038/nrurol.2014.135, DOI	2014	11.73	**2016**	2019	
Sharp VJ, 2013, AM FAM PHYSICIAN, V88, P835	2013	9.89	**2016**	2018	
Ramachandra P, 2015, WEST J EMERG MED, V16, P190, DOI 10.5811/westjem.2014.11.22495, DOI	2015	8.09	**2016**	2020	
Parlaktas BS, 2014, ASIAN J ANDROL, V16, P314, DOI 10.4103/1008-682X.122202, DOI	2014	7.44	**2016**	2019	
Sheth KR, 2016, J UROLOGY, V195, P1870, DOI 10.1016/j.juro.2016.01.101, DOI	2016	10.07	**2017**	2022	
Mestrovic J, 2014, UROLOGY, V84, P1194, DOI 10.1016/j.urology.2014.07.022, DOI	2014	7.94	**2017**	2019	
Lian BSY, 2016, EUR J PEDIATR SURG, V26, P17, DOI 10.1055/s-0035-1566096, DOI	2016	7.66	**2017**	2022	
Shimizu S, 2016, INT J UROL, V23, P454, DOI 10.1111/iju.13110, DOI	2016	14.7	**2018**	2022	
Pogorelic Z, 2016, CAN J UROL, V23, P8594	2016	16.6	**2019**	2022	
Mellick LB, 2019, PEDIATR EMERG CARE, V35, P821, DOI 10.1097/PEC.0000000000001287, DOI	2019	11.91	**2019**	2022	
Bandarkar AN, 2018, PEDIATR RADIOL, V48, P735, DOI 10.1007/s00247-018-4093-0, DOI	2018	10.35	**2019**	2022	
Arena S, 2017, EXP THER MED, V13, P2115, DOI 10.3892/etm.2017.4289, DOI	2017	9.36	**2019**	2022	
Howe AS, 2017, TRANSL ANDROL UROL, V6, P1159, DOI 10.21037/tau.2017.09.10, DOI	2017	9.3	**2019**	2022	
Grimsby GM, 2018, UROLOGY, V116, P150, DOI 10.1016/j.urology.2018.03.009, DOI	2018	8.78	**2019**	2022	
Shokoohi M, 2018, ANDROLOGIA, V50, P0, DOI 10.1111/and.13047, DOI	2018	8.78	**2019**	2022	
Bayne CE, 2017, J PEDIATR-US, V186, P200, DOI 10.1016/j.jpeds.2017.03.037, DOI	2017	8.26	**2019**	2022	
Jacobsen FM, 2020, WORLD J MENS HEALTH, V38, P298, DOI 10.5534/wjmh.190037, DOI	2020	10.77	**2020**	2022	
Macdonald C, 2018, J PEDIATR UROL, V14, P503, DOI 10.1016/j.jpurol.2018.09.017, DOI	2018	9.06	**2020**	2022	
Dejban P, 2019, THERIOGENOLOGY, V140, P136, DOI 10.1016/j.theriogenology.2019.08.021, DOI	2019	7.92	**2020**	2022	

Figure S1 The top 30 cited references sorted by the beginning year of burst. References marked in warm colors indicated a sudden increase in the usage frequency of this reference during that period. Cool colors represented a relatively unpopular time period.
